# Predictors of non-adherence to cervical cancer screening among immigrant women in Ontario, Canada

**DOI:** 10.1016/j.pmedr.2023.102524

**Published:** 2023-11-22

**Authors:** Kayla A. Benjamin, Nina Lamberti, Martin Cooke

**Affiliations:** a Institute of Health Policy, Management and Evaluation, University of Toronto, Toronto, ON, Canada; b Dalla Lana School of Public Health, University of Toronto, 155 College St 6th Floor, Toronto, ON M5T 3M6, Canada; c School of Public Health Sciences, Faculty of Health, University of Waterloo, 200 University Ave W, Waterloo, ON N2L 3G5, Canada; d Department of Sociology and Legal Studies, Faculty of Arts, University of Waterloo, 200 University Ave W, Waterloo, ON N2L 3G5, Canada

**Keywords:** Cervical cancer, Papanicolaou test, Screening, Adherence, Immigrant health, Women’s health

## Abstract

Cervical cancer is one of the most common types of cancer among women and is largely preventable with regular screening using Papanicolau (Pap) tests. In Canada, all provinces have regular screening programs, although with slightly differing recommendations. Previous research has found that immigrant women, who are a large proportion of the Canadian population, are at higher risk of being under-screened, or non-adherent to the recommended screening frequency.

Using data from the 2017 Canadian Community Health Survey, this study examined: (1) the extent to which immigration status and time since immigration are associated with Pap test adherence in Ontario, and (2) predictors of Pap test adherence for immigrants and Canadian born populations in Ontario, Canada’s most populous province, with a focus on the role of racial or ethnic identity among immigrants. Estimates of 3-year test adherence were 71.3 % (95 %CI: 66.9–75.7) among immigrant women and 75.4 % (95 %CI: 73.1–77.1) among non-immigrant women. Recent immigrants (6–10 years in Canada) had lower adherence (63.5 %, 95 %CI: 48.0–80.0). Logistic regression models found that immigrant women had lower adherence than Canadian-born women, controlling for age, household income, education, and having a primary care physician. Subgroup analysis found that South Asian immigrant women were least likely to be adherent.

These results support targeted programming to increase screening adherence among recent immigrants and raise concerns regarding potential barriers to screening. Data that allow better disaggregation of racial and ethnic identities are important for better understanding the potential implications of these patterns for racial inequities in health.

## 1 Introduction

Globally, cervical cancer has the fourth-highest incidence of all types of cancer among women (Sung et al., 2021). It is estimated that, in 2022, 1,450 cervical cancer diagnoses and 380 deaths will have occurred among Canadians (Canadian Cancer Statistics Advisory et al., 2022). Despite its high incidence, cervical cancer is largely preventable with regular Papanicolaou (Pap) tests (Safaeian et al., 2007, Skinner et al., 2016). The Pap test is a cervical swab used to collect a sample of cells. With regular Pap tests, cervical cancer is often diagnosed at a precancerous or early stage (Ontario, 2020, Franco et al., 2001).

Many high-income countries (HIC) have implemented Pap test screening programs since the middle of the last century, however in recent years some have begun adopting human papilloma virus (HPV) testing as the primary screening modality (Bhatla and Singhal, 2020). In Canada, the Pap test continues to be used as the primary modality in all provinces and territories except for Quebec and Prince Edward Island, both of which changed to HPV testing in 2022 and 2023, respectively (Aziz, 2023).

Ontario is working to implement HPV testing; however, the current recommendation is for individuals with a cervix aged 21 to 70, who are or who have been sexually active, to obtain a Pap test once every three years (Cancer Care Ontario, 2020). Prior to 2012, Ontario recommended annual screening (Public Health Ontario, 2017). Those who are eligible may receive letters from the Ontario Cervical Screening Program (OCSP) to remind them to book a test. Individuals with family doctors will be informed during annual physicals whether they are due for a Pap test (Cancer Care Ontario, 2020).

Despite OCSP letters and the availability of free screening for those covered under the Ontario Health Insurance Plan, there are still many eligible people who do not adhere to the provincial screening guidelines. However, this risk of under-screening is not uniformly distributed. Previous studies have found that immigrant women were more likely to have never been screened or be underscreened for cervical cancer than Canadian-born women (Bacal et al., 2019, Datta et al., 2018, Lee et al., 2019, Lofters et al., 2010, Voruganti et al., 2016). Notably, a study by Datta et al. (2018) using several cycles of the Canadian Community Health Survey (CCHS) (2003, 2005, 2008, and 2012) to explore screening in Montreal found that immigrant status was the strongest predictor of having never received a Pap test, with prevalence ratios ranging from 2.7 (95 % CI: 2.1–3.5) for women who immigrated more than 10 years ago to 3.9 (95 % CI: 2.9–5.4) for women who had immigrated within the previous five years. Other studies have reported similar findings, in which recent immigrants were at a greater risk of not being screened compared to longer-term immigrants (Woltman and Newbold, 2007).

As Canada is a high-immigration country, immigrants to Canada are a large and important population for public health and health promotion efforts. In the 2021 census, 23.0 % of Canadians indicated that they were immigrants, and this figure was 30 % in Ontario, the province in which 44 % of recent arrivals to Canada were living (Statistics Canada, 2022). More than 400,000 immigrants are expected to arrive in Canada in 2023–24 (Immigration, Refugees and Citizenship Canada, 2023).

In Canada, immigration, and racial and ethnic inequalities are closely related. Since fundamental changes to the Canadian immigration system ended legal racial discrimination in immigration, Canada became open to immigration from non-European and non-American countries in 1962. In 1967, race and nationality were excluded from being explicit parts of the point system that ranked the eligibility of potential economic immigrants (Troper, 1993). In 2021, 62.0 % of the most recent immigrants in Canada reported having been born in Asia, including the Middle East, and another 15.6 % had been born in Africa (Statistics Canada, 2022). Any systemic barriers to health service utilization by immigrants will potentially contribute to racial differences in health outcomes, which are themselves an important public health policy issue in Canada (Bhopal, 2007).

There is some evidence to support a relationship between race and ethnicity and adherence to regular cervical cancer screening, although the studies are few (Nnorom et al., 2019). An earlier study by McDonald and Kennedy (2007) using cycles of the National Population Health Study (2000–01) and CCHS (2002–03), found that racialized immigrants had lower rates of Pap test adherence than white immigrants from English-speaking backgrounds. Several studies found that immigrants to Canada from Asia or South Asia were at particular risk for being under-screened (Maxwell et al., 2001, Lofters et al., 2010, Lee et al., 2019).

Due to the dynamic nature of the Canadian immigration system, including the changing composition of immigration flows, and changes to the provincial screening programs, timely information regarding immigrants’ interactions with the healthcare system is important. The CCHS is the most consistent national source of data for monitoring health-related behaviours in Canada. However, the latest use of this survey to examine the issue of Pap screening among immigrants used data from 2007 to 2016 (Bacal et al., 2019), when Ontario still recommended annual screening. Since that time, the CCHS has undergone a redesign, implemented for the 2015 cycle, including a new collection strategy and content revisions (Thomas and Tremblay, 2020) with significant changes to questions regarding Pap tests. A more recent study that did include the 2016 CCHS cycle, evaluated screening adherence across CCHS datasets from 2007 to 2016; however, this study looked at the whole Canadian sample, which did not account for varying provincial guidelines or the CCHS redesign (Abdel-Rahman, 2021).

Using more recent data from the CCHS (2017 cycle), this study aimed to (1) investigate the extent to which immigration status and time since immigration are associated with Pap test adherence in Ontario, and (2) examine the predictors of Pap test adherence for immigrants and Canadian born populations in Ontario, with a focus on the role of racial or ethnic identity among immigrants.

## 2 Methods

The analysis used the 2017 CCHS, collected by Statistics Canada and accessed through the Research Data Centres Program.^1^ The CCHS is an annual survey that collects health and health behaviour data from Canadians aged 12 years and older living outside of First Nations reserves and institutionalized settings (Statistics Canada, 2018). Data were collected using computer-assisted telephone and computer-assisted personal interviewing, in the language (official or non-official) of the respondent’s choice. The survey samples roughly 65,000 Canadians annually (Statistics Canada, 2018). This study used anonymized publicly available data and therefore was not subject to institutional ethics review.

Our outcome measures were “adherence” with provincial screening guidelines. To align with Ontario’s Pap test recommendations the analysis sample was limited to respondents aged 21–69 and living in Ontario (Cancer Care Ontario, 2020). The survey asked respondents identifying as female at birth who had not had a hysterectomy, whether they had “ever had a Pap smear test”. Those reporting positively were then asked when they had last received a test and how often they normally receive a test. A complete list of possible responses is available in Supplementary Material.

From these variables we created two binary outcome variables. The first indicated whether the respondent had had a Pap test within the preceding three years and the second indicated whether they reported generally having a test at least once every three years. In both cases, we coded respondents as either “adherent” or “non-adherent” according to the provincial guidelines. The small number of respondents indicating they “did not know” were removed. As with previous papers using CCHS data (Datta et al., 2018, Bacal et al., 2019), these estimates include women who were not sexually active, and should therefore be considered underestimates of true adherence to the guidelines.

The CCHS also asks respondents whether they are (or have been) immigrants to Canada, the time since their immigration, as well as other socio-demographic characteristics including educational attainment and total household income before taxes. These were recoded into broad categories for analysis. Respondents are asked to self-report the “racial or cultural” group(s) to which they belong, with the question presenting a list of 12 possible responses as well as “Other (specify).” Rather than combine large and small categories based off geographical proximity we retained the largest categories (“White”, “Chinese”, “South Asian” “Black” and “Filipino”) and combined, “Latin American”, “Arab”, “Southeast Asian”, “West Asian”, “Korean”, “Japanese” and “other” as “Other.” Although we recognize the ethnic and cultural differences between categories, these categories were amalgamated due to small sample sizes. Those who provided multiple responses were coded as “multiracial.” Importantly for our purposes, the survey asks whether respondents had a family doctor, which we expect is a significant predictor of adherence to provincial screening guidelines. This was included as a binary variable.

### 2.1 Statistical Analyses

In an exploratory data analysis we first compared the distributions of the predictor variables for immigrant and Canadian-born women using chi-square tests of independence. We then estimated the unadjusted percentages of immigrant and non-immigrant women who were “adherent” to the Ontario guidelines in 2017–18, reporting percentages and 95 % confidence intervals.

We then used binary logistic regression to model the association between immigrant status and adherence, adjusting for age, as well as education, total household income, self-rated health and whether the respondent had a family doctor. As described above, previous research has found that racialized women in Canada are less likely to receive cervical cancer screening at the recommended intervals. A key interest of ours was whether racialized immigrants were less adherent to guidelines than were non-racialized immigrants. We therefore conducted a subgroup analysis in which we limited the sample to immigrants (n = 1074) and included the racial/cultural variable as well as age group, income, education, self-rated health and whether the respondent had a primary care physician.

Models were estimated using the bootstrap weights provided on the dataset and the procedure recommended by Statistics Canada, to adjust the estimates of variance for the complex (clustered and stratified) nature of the sample (Gagné et al., 2014). Analyses were conducted using SAS v. 9.4 (SAS Institute, Cary, N.C.).

## 3 Results

Table 1 presents the distributions of the main independent variables by immigration status, our main predictor variable. The total unweighted sample size was 5,043 where 21.3 % (n = 1,074) were immigrants and 78.7 % (n = 3,969) were Canadian born women. We are not able to report distributions of racial categories by immigration status due to small cell sizes and Statistics Canada guidelines.

**Table 1:** Distributions of Independent variables (percent) by immigration status, women aged 21–69, Ontario Canada, 2017.

	Immigrant (%) (n = 1,074)	CanadianBorn (%) (n = 3,969)	*Χ ^2^* Significance
*Age Group*			*<0.0001*
21–29	9.33	21.09	
30–39	24.37	20.87	
40–49	22.99	19.57	
50–59	24.70	20.89	
60–69	18.61	17.58	
*Total Household Income*			*<0.0001*
No income–$19,000	6.48	7.72	
$20,000–$59,999	26.03	21.15	
$60,000–$99,999	23.21	22.71	
$100,000–$149,999	19.59	20.15	
≥$150,000	24.69	28.27	
*Highest Educational Attainment*			*<0.0001*
Less than secondary school	6.92	4.63	
Secondary school, no post-secondary	18.68	20.03	
Post-secondary certificate, diploma, or degree	74.39	75.34	
*Reported having a Primary Care Physician*			*<0.0001*
Yes	92.17	91.07	
No	7.83	8.93	
*Self-Rated Health Status*			*<0.0001*
Poor	2.19	2.69	
Good/Fair	39.06	31.67	
Excellent	58.75	65.64	
*Marital Status*			*<0.0001*
Married/Common Law	68.68	61.37	
Widowed/Separated/Divorced	12.01	11.77	
Single/Never Married	19.31	26.86	

*Note*: Unweighted sample sizes (n) shown. Population weights have been applied to calculate percentages,

Data Source: 2017 Canadian Community Health Survey.

In Table 2 we present estimated percentages of the Ontario population reporting adherence (a Pap test at least every three years), by immigration status and time in Canada. Immigrants were generally less likely than Canadian born women to adhere to this guideline. As shown in Table 2, 71.28 % (95 %CI: 66.89–75.67) of immigrants reported “usually” receiving screening every three years compared to 75.42 % (95 %CI: 73.12–77.70) of Canadian born women. Similarly, when examining those tested within the previous three years, only 73.32 % (95 %CI: 68.99–77.64) of immigrant women were found to be adherent compared to 77.46 % (95 %CI: 69.07–77.71) of Canadian born women. Recent immigrants were the least likely to be adherent, by either measure. Among those who had been in Canada from six to ten years, only 63.5 % reported having been screened in the previous three years (95 %CI: 48.01– 78.98) and 62.90 % reported that they were usually tested every three years (95 %CI: 47.41–78.38). Among those in Canada less than five years, roughly half were adherent by either measure, although it should be noted that this category includes women who had not yet been in Canada for three years at the time of the survey.

**Table 2:** Percentage adherent to provincial screening guidelines by immigrant status and time since immigration, women aged 21–69, Ontario Canada, 2017.

	Adherent (last Pap within 3 years)		Adherent (“usually” tested every three years)	
	%	95 %CI	%	95 %CI
Immigrants (0–5 years)	50.20	35.32–65.08	48.06	33.18–62.93
Immigrants (6–10 years)	63.50	48.01– 78.98	62.90	47.41–78.38
Immigrants (≥ 11 years)	78.59	74.35–82.83	76.32	71.94–80.69
Immigrant total (n = 1,074)	73.32	68.99–77.64	71.28	66.89–75.67
Canadian-born (n = 3,969)	77.46	69.07–77.71	75.42	73.11–77.70

Table 3 presents regression models predicting adherence, while adjusting for age group, income, education, self-rated health, and whether respondents had a primary care physician. For both outcomes, immigrants had significantly lower odds of being adherent to the provincial screening guidelines of a test every three years, controlling for the other variables in the model. The odds of an immigrant woman having had a Pap test within the previous three years was 0.69 that of a non-immigrant woman (95 %CI: 0.55–0.89). The odds ratio for reporting that they “usually” had a test every three years was similar (OR: 0.72, 95 %CI: 0.57–0.92).

**Table 3:** Logistic Regression models of adherence to provincial Pap screening guidelines, among Immigrant women (n = 1,074) and Canadian women (n = 3,969) Ontario, Canada, 2017.

	Last Pap within 3 yearsAdjusted OR (95 %CI)	Usually tested ≤ 3 yearsAdjusted OR (95 %CI)
*Immigrant status*		
Immigrant	0.69 (0.55–0.89)**	0.72 (0.57–0.92)**
Canadian born (ref)	–	–
*Age Group*		
21–29	0.42 (0.29–0.60)**	0.43 (0.30–0.61)**
30–39 (ref)	–	–
40–49	0.95 (0.65–1.38)	0.92 (0.65–1.31)
50–59	1.03 (0.72–1.47)	0.95 (0.68–1.33)
60–69	0.62 (0.42–0.86)*	0.59 (0.42–0.83)**
*Total Household Income*		
No income–$19,999	1.02 (0.62–1.63)	1.05 (0.67–1.66)
$20,000–$59,999	0.88 (0.64–1.20)	0.88 (0.65–1.19)
$60,000–$99,999 (ref)	–	–
$100,000–$140,999	1.17 (0.83–1.65)	1.23 (0.88–1.72)
≥$150,000	1.21 (0.85–1.73)	1.37 (0.97–1.93)
*Highest Educational Attainment*		
Less than secondary school	0.35 (0.22–0.57)**	0.40 (0.25–0.64)**
Secondary, no post-secondary	0.59 (0.43–0.79)**	0.64 (0.49–0.86)**
Post-secondary certificate, diploma, or degree (ref)	_	_
*Reported having a Primary Care Physician*		
Yes (ref)	_	_
No	0.32 (0.21–0.48)**	0.36 (0.24–0.54)**
*Self-Rated Health Status*		
Poor	0.86 (0.49–1.51)	0.92 (0.53–1.59)
Good / Fair	0.84 (0.65–1.08)	0.84 (0.66–1.06)
Excellent (ref)	_	_
*Marital Status*		
Married / Common Law (ref)	_	_
Widowed / Separated / Divorced	0.95 (0.70–1.29)	1.02 (0.76–1.34)
Single / Never Married	0.60 (0.45–0.89)**	0.66 (0.49–0.88)**

*: significant at P ≤ 0.05, **: significant at P ≤ 0.01.

Independent of immigration status, there were few significant predictors of adherence. For both outcomes, those in the youngest age range (21–29), and the oldest range (60–69) had significantly lower odds of adherence than those aged 30 to 39 (Table 3). Those without a primary care physician were at higher risk of non-adherence: the odds of having had a Pap test within three years were 0.32, relative to those with a primary care physician (95 %CI: 0.21–0.48). The odds ratio for the second outcome (usually getting tested every three years) was similar (OR: 0.36, 95 %CI: 0.24–0.54). Education was also a significant independent predictor of adherence. Those with less than a post-secondary education were significantly less likely to adhere to screening guidelines for both outcomes (Table 3). Finally, respondents who were single or never married were less likely to have had a Pap test within the past three years, relative to married or partnered respondents (OR: 0.60, 95 %CI: 0.45–0.89). The odds ratio for the second outcome (usually getting tested every three years) was similar (OR: 0.66 95 %CI: 0.49–0.88).

Income category and self-rated health status were not found to be independent predictors of either outcome.

**Fig. 1:**
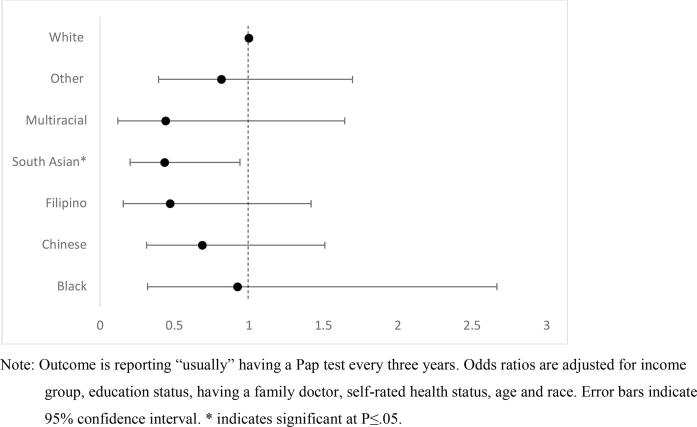
Odds Ratios of Pap test adherence among women immigrants aged 21–69 in Ontario, Canada (N = 1074), by racial-cultural identity, 2017. Note: Outcome is reporting “usually” having a Pap test every three years. Odds ratios are adjusted for income group, education status, having a family doctor, self-rated health status, age and race. Error bars indicate 95 % confidence interval. * indicates significant at P ≤ 0.05.

As described above, we conducted a subgroup analysis of the sample of immigrant women to examine the independent association of race and adherence among immigrants. Here, “white” immigrants were coded as the reference value because it was the largest sub-category. The models produced similar results to the initial analysis, in terms of the direction and significance of the independent variables. Immigrants in the youngest and oldest age ranges, as well as those without a primary care physician, had significantly lower odds of adherence to screening guidelines. Among immigrants, the racial-cultural identity variable did identify groups at risk. Fig. 1 shows the adjusted odds ratios for “usually” having a Pap test every three years, with confidence intervals. Women identifying as South Asian (OR: 0.44, 95 %CI: 0.20–0.94) were significantly less likely to report adherence by this measure. Those reporting multiracial background, Filipino or Chinese ethnicity had lower estimated odds of adherence than white women, although not significant at the 0.05 level. The odds of adherence for Black immigrant women and those identifying “other” background were most similar to white immigrant women, possibly reflecting the heterogeneity of these groups in terms of cultural backgrounds and countries of origin. The results for the model predicting having had a Pap test within the previous three years were similar (results not shown).

## 4 Discussion

Our analysis found that ≈ 27 % of immigrant women in Ontario have not been screened for cervical cancer within the last three years and ≈ 29 % are not “usually” adherent to provincial screening guidelines of being tested at least once every three years. While this is not statistically different from non-adherence rates among Canadian-born women in Ontario ≈ 23 % not screened in the last three years and ≈ 25 % not usually adherent, this demonstrates that there remains an important percentage of immigrant women who are not receiving appropriate screening for cervical cancer.

Our study is the first, using CCHS data, to estimate the percentage of non-adherent immigrant and Canadian-born women in Ontario since provincial guidelines changed regarding screening frequency. Prior to 2012, women living in Ontario were recommended to obtain a Pap test once every year. Using 2012 CCHS data, Bacal et al. (2019) estimated that 30 % of immigrant women were underscreened compared to 19 % of Canadian-born women, nationally.

Since 2013, the recommended frequency of screening in Ontario is once every three years (Cancer Care Ontario, 2020). Having ≈ 29 % of immigrants not regularly accessing Pap tests in Ontario, despite this change in guidelines, demonstrates that there is a continued need to focus on this population in the development of public health and health promotion efforts for cervical cancer screening.

Age and having a primary care physician were important predictors of adherence. There is a strong body of international evidence showing that older age (Bacal et al., 2019) and not having a primary care physician is associated with lower screening adherence, which is supported by our findings (Deguara et al., 2021, Okolie et al., 2021, Worthington et al., 2012, Yimer et al., 2021). However, few studies have highlighted the higher vulnerability of younger adult women to underscreening (Lofters et al., 2011, Rodvall et al., 2005), as we found. In Ontario, this may be partially explained by the eligibility requirement stipulating that women must be or have previously been sexually active to obtain a Pap test. The Annual Component of the CCHS does not ask women about their sexual activity.

We find a clear association between adherence and time in Canada. In our sub-analysis, focused on predictors of adherence among immigrant women, ≈ 48 % of recent immigrants were adherent compared to ≈ 76 % of immigrants who had been in Canada for 11 years or more. This finding is consistent with other studies that have found recent immigrants were more vulnerable to underscreening (Datta et al., 2018, Lofters et al., 2010, Xiong et al., 2010). Our study confirms this association with updated CCHS data and in the context of the newer provincial screening guidelines.

Acculturation is often cited as an explanation of this trend. Studies have found that measures of acculturation are positively related to Pap test screening among South Asian women who immigrate to Canada (Brotto et al., 2008, Gupta et al., 2002). However, in a high-immigration country, such as Canada, public health practitioners should target barriers to screening for immigrants, including cultural, language, healthcare system-related, and knowledge-related barriers (Ferdous et al., 2018).

Unsurprisingly, we found that immigration status and racial or cultural identity were highly correlated, such that including both variables in a single model was untenable. Immigrant women being significantly less likely to adhere to screening guidelines may contribute to racial inequities in cervical cancer incidence in Canada (Simkin et al., 2021). Our sub-analysis explored differences in adherence among immigrants by racial-cultural identity. However, small sample sizes restricted our ability to disaggregate this data. Previous studies attempting to assess this relationship have relied on the same CCHS variables included in our study (Datta et al., 2018, Schoueri-Mychasiw and McDonald, 2013) or have used country of origin as a proxy for racial or ethnic identity (Maxwell et al., 2001), neither of which is sufficient for fully unpacking the impact of race and ethnicity on screening adherence. There is an important need for Canada to collect ethical and culturally appropriate race-based data (El-Mowafi et al., 2021).

Regardless, our sub-analysis of immigrant women did find that women identifying as South Asian were significantly less likely than the reference, “white” immigrants, to report adhering to screening guidelines. While more research is needed, religiosity and conservative feelings toward sex could, in part, explain underscreening.

While our study is focused in Ontario, Canada, these findings have important relevance for other HIC and high-immigration countries. As HIC adopt HPV tests as a primary screening modality for cervical cancer (Bhatla and Singhal, 2020), it is important that they first understand who in their population is at risk for underscreening and what socio-demographic and health system factors are associated with underscreening. This understanding is critical for ensuring eligible individuals can and will access HPV tests.

## 5 Limitations

The study has several limitations. First, it is limited by the use of the CCHS self-reported survey data. Meaning our study might be subject to recall and misclassification bias. Lofters et al. (2015) found that there is a pervasive tendency for people in Ontario to over-report their screening histories. Further, immigrants appear to be under-represented in the CCHS data. The 2016 census reported that 34.5 % of women aged 15 and older in Ontario were immigrants (Statistics Canada, 2017), while 21.3 % of our analysis sample of women aged 21 to 69 were immigrants. As the CCHS is a voluntary survey, it could be that immigrants who chose not to respond to the survey were also less likely to be adherent to screening guidelines, potentially biasing our estimations of adherence upwards. The relationship between the potential underrepresentation of immigrants and our independent or dependent variables is unknown, however. Despite these limitations, the CCHS remains one of the most comprehensive data sets available that includes immigrant status and adherence to cervical cancer screening.

Due to small cell sizes and Statistics Canada’s rules for disclosure, we were not able to report on the distributions of racial-cultural categories, as described above. While we could have increased cell sizes by considering more than one province or adding additional CCHS cycles, this would have produced new limitations. Pooling immigrant populations across multiple provinces and territories would be challenging due to differences in screening guidelines and that not all CCHS cycles collect data on Pap tests for all provinces and territories.

## 6 Conclusion

Our findings suggest that targeted public health interventions are needed to increase Pap test adherence among structurally vulnerable communities in Ontario, specifically recent immigrants, and racialized communities. To this end, public health might work with multicultural organizations to develop culturally specific resources, considerate of different religious values, and target immigrant and racialized communities in Ontario. These agencies could play a critical role in reaching recent immigrants to inform and encourage them to obtain regular Pap tests, especially for those who do not currently have a primary care physician. Finally, further research is needed to interrogate the impact of race, ethnicity, and immigration on screening adherence. This would require large data sets with disaggregated race data. Considering the limitations of self-reported screening data, this is best generated by linking administrative data on cancer screening to immigration and race-based data that is collected in an ethical and culturally appropriate way.

## CRediT authorship contribution statement

**Kayla A. Benjamin:** Conceptualization, Methodology, Writing – original draft. **Nina Lamberti:** Formal analysis, Writing – original draft. **Martin Cooke:** Conceptualization, Methodology, Writing – review & editing, Supervision.

## Declaration of Competing Interest

The authors declare that they have no known competing financial interests or personal relationships that could have appeared to influence the work reported in this paper.

## Data Availability

The authors do not have permission to share data.

## Footnotes

1 This research was supported by funds to the Canadian Research Data Centre Network (CRDCN) from the Social Science and Humanities Research Council of Canada, (SSHRC), the Canadian Institutes for Health Research (CIHR), the Canadian Foundation for Innovation (CFI) and Statistics Canada. Although the research and analysis are based on data from Statistics Canada, the opinions expressed do not represent the views of Statistics Canada or the Canadian Research Data Centre Network (CRDCN).
