# Supplementary material for: Predictors of non-adherence to cervical cancer screening among immigrant women in Ontario, Canada
Source: Prev Med Rep. 2023 Nov 22;36:102524. doi: 10.1016/j.pmedr.2023.102524 (PMC10728462; doi:10.1016/j.pmedr.2023.102524)
Supplement: Supplementary Data 1 [file mmc1.docx]

**Supplementary Material**

Table A.1: List of all possible responses to CCHS Pap Smear test questions

| ***When was the last time you received a Pap Smear test?*** | ***How often do you usually receive a Pap Smear test?*** |
| --- | --- |
| Less than one year to one year ago | It was the first time |
| More than 1 year to 2 years ago | More than once a year |
| More than 2 yeas to 3 years ago | Between once a year to less than every 3 years |
| More than 2 years to 5 years ago | Every three years |
| More than 5 years ago | Less often than every 3 years |
|  | No fixed frequency |
